# Defining Melanoma Immune Biomarkers—Desert, Excluded, and Inflamed Subtypes—Using a Gene Expression Classifier Reflecting Intratumoral Immune Response and Stromal Patterns

**DOI:** 10.3390/biom14020171

**Published:** 2024-01-31

**Authors:** Agata Mlynska, Jolita Gibavičienė, Otilija Kutanovaitė, Linas Senkus, Julija Mažeikaitė, Ieva Kerševičiūtė, Vygantė Maskoliūnaitė, Neda Rupeikaitė, Rasa Sabaliauskaitė, Justina Gaiževska, Karolina Suveizdė, Jan Aleksander Kraśko, Neringa Dobrovolskienė, Emilija Paberalė, Eglė Žymantaitė, Vita Pašukonienė

**Affiliations:** 1 National Cancer Institute, LT-08406 Vilnius, Lithuania; jolita.gibaviciene@nvi.lt (J.G.); otilija.kutanovaite@nvi.lt (O.K.); rasa.sabaliauskaite@nvi.lt (R.S.); neringa.dobrovolskiene@nvi.lt (N.D.); e.paberale@gmail.com (E.P.); vita.pasukoniene@nvi.lt (V.P.); 2 Faculty of Fundamental Sciences, Vilnius Gediminas Technical University, LT-10223 Vilnius, Lithuania; 3 Life Sciences Center, Vilnius University, LT-01513 Vilnius, Lithuanianeda.rupeikaite@gmc.stud.vu.lt (N.R.); 4 National Center of Pathology, LT-08406 Vilnius, Lithuania

**Keywords:** melanoma, tumor-infiltrating lymphocytes, biomarkers, immune subtypes, desert, excluded, inflamed, gene expression, tumor microenvironment, classifier, TCGA

## Abstract

The spatial distribution of tumor infiltrating lymphocytes (TILs) defines several histologically and clinically distinct immune subtypes—desert (no TILs), excluded (TILs in stroma), and inflamed (TILs in tumor parenchyma). To date, robust classification of immune subtypes still requires deeper experimental evidence across various cancer types. Here, we aimed to investigate, define, and validate the immune subtypes in melanoma by coupling transcriptional and histological assessments of the lymphocyte distribution in tumor parenchyma and stroma. We used the transcriptomic data from The Cancer Genome Atlas melanoma dataset to screen for the desert, excluded, and inflamed immune subtypes. We defined subtype-specific genes and used them to construct a subtype assignment algorithm. We validated the two-step algorithm in the qPCR data of real-world melanoma tumors with histologically defined immune subtypes. The accuracy of a classifier encompassing expression data of seven genes (immune response-related: *CD2*, *CD53*, *IRF1*, and *CD8B*; and stroma-related: *COL5A2*, *TNFAIP6*, and *INHBA*) in a validation cohort reached 79%. Our findings suggest that melanoma tumors can be classified into transcriptionally and histologically distinct desert, excluded, and inflamed subtypes. Gene expression-based algorithms can assist physicians and pathologists as biomarkers in the rapid assessment of a tumor immune microenvironment while serving as a tool for clinical decision making.

## 1. Introduction

Emerging cancer immunology discoveries reshaped the way we perceive tumors through the point of view of the host’s immune system. The proactive antitumor immune response, manifested by the functional antigen recognition and infiltration of tumor tissue by activated T cells is often counterbalanced by the quick adaptation of the tumor microenvironment (TME) [1]. Although the dynamics of cancer-immunity interactions unveil another level of complexity on top of these two already elaborate subjects [2,3], some principal phenomena were successfully translated into clinical cancer management at the diagnostic (e.g., Immunoscore [4]), monitoring (e.g., baseline local and circulating biomarkers [5]), and, in particular, therapeutic (e.g., immunotherapy application [6]) levels.

However, the efficacy of immunotherapy is often hampered due to the lack of a personalized approach and limited understanding of the tissue-specific immune contexture [7]. The heterogeneity of responses encourages the development of large-scale molecular data-based algorithms that would allow for the identification of target patient populations for personalized immunotherapy solutions [8,9]. With the accumulation of big data input, efforts are being made to extract clinically relevant information from large datasets and repositories [10]. Nevertheless, the need for a clear and easy TME classification remains.

One of the proposed tumor immune subtyping strategies, distinguishing desert, excluded, and inflamed subtypes, emerged from observing distinct tumor histological patterns and responses to a checkpoint blockade [11,12,13]. This classification system considers the presence and location of tumor-infiltrating leukocytes (TILs). Desert tumors are poorly immune-infiltrated and lack the pre-existing antitumor immunity (no TILs). Excluded tumors have a dense extracellular matrix and retain TILs in the reactive stroma. Inflamed tumors are characterized by considerable infiltration of TILs, often not properly functioning. Although universal in nature and potentially compliant as an immunotherapy biomarker, this subtyping system still needs more evidence to reach consensus in the academic and clinical network.

We have previously identified a gene signature for immune subtyping of high-grade serous ovarian cancer [14]. In this study, we took advantage of the clinical and RNA-Seq data of the cutaneous melanoma dataset deposited in The Cancer Genome Atlas (TCGA) and explored the immune desert, excluded, and inflamed subtype-specific transcriptional patterns. We extracted the top differentially expressed genes and constructed a seven-gene classifier for assigning the patients into immune subtypes. The performance of the classifier was validated in a real-world patient cohort and compared to histological tumor profiles.

## 2. Materials and Methods

### 2.1. Processing of TCGA Dataset and Gene List

The full clinical and level 3 RNA-Seq transcriptomic data of 480 available melanoma patients in the TCGA-SKCM database [15] were retrieved through cBioportal web API [16,17] on 7 January 2022. Data were preprocessed to remove entries containing empty or duplicate values, which resulted in withdrawing 20 clinical entries.

The immune response- and stroma-related genes were selected based on previously published studies and profiling panels [18,19], resulting in a list of 1578 genes for further analysis. After calculating the coefficient of variation for each selected gene expression in the TCGA dataset, 201 genes with low dispersion (CV < 5%) and 3 genes with missing values were eliminated from further analysis. No outliers were detected using hierarchical clustering or principal component analysis. As a result, the final TCGA training dataset consisted of 1372 gene expression scores from 460 patients with melanoma tumors.

### 2.2. In Silico Immune Subtyping

We next performed cluster analysis to explore the immune response-related transcriptional patterns independent of their histological phenotype. The optimal number of clusters was determined based on elbow (distortion) [20] and silhouette [21] methods using Python package *Yellowbrick* v1.5 [22]. With regard to reports on tumor immune typing and immunotherapy biomarkers [14,18,23], we empirically selected forty genes from a 1372 gene list to serve as the determinants for clustering the TCGA cohort into subtypes using *k*-means [24] or a previously published predefined rule coefficient calculation algorithm [14] that requires grouping the selected genes into angiogenesis, stroma, and immune response categories, which are prominent in immune desert, exclusion, and inflammation states, respectively. Only those clinical TCGA records that were assigned to the same immune subtype group by both clustering methods were then selected for further differential gene expression analysis and classifier modeling.

### 2.3. Differential Gene Expression Analysis and Building Subtyping Classifier

The normalized and filtered RSEM [25] read counts from TCGA were processed with R package *limma* for differential gene expression analysis using linear regression model, which tests for gene expression dependence on immune subtype using formula ∼0 *+ Subtype*, where zero is the logistic regression intercept and allows for comparison between different subtypes [26]. Contrasts created with such a formula can be adjusted to reflect differences between immune subtypes. A design matrix consisting of patient identifiers, assigned immune tumor subtypes, and contrasts was created. The design matrix and gene expression estimates were analyzed with *ImFi* method in *limma* via weighted least squares estimation. The obtained results were used to evaluate the expression of each gene in different immune subtypes with *contrasts.fit* and *eBayes* methods. For each subtype, the top 10 genes with the most differential expression among all subtypes (lowest *p*-values) were selected as representative genes for further construction of the subtyping classifier.

To address the different data acquisition methods in training TCGA dataset (RNA-Seq) and real-world patient validation cohort (qPCR), we settled for the z-transformation of gene expression data as a well-performing means for scoring samples against multiple molecular signatures [26]. To improve the robustness and better control for outliers, we calculated the modified z-scores utilizing the median and median absolute deviation [27].

We then combined several gene expression biomarkers to achieve the optimal power to discriminate the melanoma immune phenotype. Using ROC analysis, the selected top 10 subtype-representative genes were examined alone and in various combinations as factors for subtype discrimination. Factors yielding the largest AUC, as well as sensitivity and specificity, were selected and combined in a manual decision tree which reflects the initial in silico subtyping results from Section 2.2 most accurately. The cutoff values resulting in the highest Youden’s index (defined as sensitivity + specificity-1) [28] were selected as thresholds to discriminate between subtypes.

### 2.4. Melanoma Patient Cohort

Ninety-six patients with confirmed melanoma diagnoses and no prior cancer or immune disorder history were involved in this study. All subjects signed the informed consent form before participation. The study was conducted in accordance with the Declaration of Helsinki. The study protocol was approved by the Board of National Cancer Institute (Vilnius, Lithuania) and Vilnius Regional Biomedical Research Ethics Committee (approval no. 158200-18-1004-501). Patients were enrolled in the study during 2018–2020. Patient follow-up continued until October 2023. Clinical data were obtained from medical records. All patients underwent surgery and received standard-of-care treatment according to the tumor stage. Specifically, patients diagnosed with stage II melanoma were placed on monitoring only, with no active intervention initiated unless disease progression was observed. Patients diagnosed with stage III-IV melanoma were stratified based on their BRAF mutation statuses. Patients with *BRAF* positive mutation received treatment with dabrafenib and trametinib, while those with *BRAF* wild type received checkpoint inhibitors. For each patient, surgical specimens of primary tumors were collected, processed, fixed in 10% neutral buffered formalin for 6–24 h and paraffin embedded in a MAGNUS Tissue Processor (Milestone Medical, Ipswich, QLD, Australia).

### 2.5. Histopathological Assessment

Three-micrometer-thick tissue sections from FFPE blocks were routinely stained with hematoxylin and eosin (H&E). The slides were scanned with the Aperio ScanScope XT Slide Scanner (Aperio Technologies, Inc., Vista, CA, USA) and analyzed using Aperio eSlideManager Version 12.4.3.8003 software by an experienced pathologist. The presence of intraepithelial and stromal TILs was evaluated by a trained pathologist and quantified according to the recommendations of [29,30]. Briefly, after selecting the areas within tumor borders, the type of inflammatory infiltrate was determined. Only mononuclear TIL infiltrate in tumor stroma and parenchyma was assessed, while necrotic areas also containing granulocytes were not taken into account. For TILs, a cutoff of 10% was adopted [31]. Tumors were defined as desert if less than 10% TILs could be detected within tumor. If the level of TIL infiltration was more than 10%, tumors were further distinguished based on the localization of TILs—if lymphocytes could be found only in stromal compartment but not the parenchyma, such tumors are classified as excluded. If TILs are detected in both stroma and the parenchyma in direct contact with cancer cells, tumors were considered to be inflamed (see pictures in Section 3.4).

### 2.6. qPCR for Gene Expression Analysis in Real-World Patient Cohort

Total RNA was extracted and purified from twenty-micrometer-thick tissue scrolls from FFPE blocks with truXTRAC FFPE total NA Ultra Kit (Covaris, Wouburn, MA, USA) according to manufacturer’s protocol using Adaptive Focused Acoustics ™ technology-based Covaris M220 ultrasonicator (Covaris) and column-based purification. Five hundred nanograms of purified RNA from each sample was reverse transcribed using Maxima First Strand cDNA Synthesis Kit (Thermo Fisher Scientific, Waltham, MA, USA) according to manufacturer’s guidelines. qPCR was performed in triplicate in Azure Cielo 3 Real-Time PCR System (Azure Biosystems, Dublin, CA, USA). In one reaction of ten microliters volume, there was 5 μL of Maxima SYBR Green qPCR Master Mix 2X (Thermo Fisher Scientific), 2.5 μL of the ten times diluted cDNA reaction product, and 2.5 μL of 0.8 μmol/mL sequence-specific forward and reverse primer mix (sequences taken from PrimerBank [32]). The reaction was started by incubating for 5 min at 95 °C, and 40 cycles of 10 s denaturing at 95 °C followed by 30 s of annealing/extension at 60 °C. Threshold cycle values were extracted from Azure Cielo 3 Manager software (Azure Biosystems) using logistic regression. Relative gene expression levels were calculated using the delta Ct relative quantitation method [33] with Pfaffl correction for PCR efficiency [34]. *RPL13A* and *GAPDH* served as reference genes. GE levels were normalized by calculating the modified z-scores.

### 2.7. Statistical Analysis

Data were analyzed and visualized using built-in features of GraphPad Prism 9 (GraphPad Software, version 9.1.1) and SPSS 25.0 (IBM) statistical software. Heatmaps were generated with Morpheus (Broad Institute) software [35]. Clinical features were compared using the χ2 test (categorical variables) and one-way ANOVA or Kruskal–Wallis test (numeric variables). The Kaplan–Meier survival curves were analyzed with a log-rank test. Odds ratios were derived from the univariate and multivariate Cox regression model and compared using the Wald test. The ROC curves and AUC were derived from sensitivity and specificity. Performance metrics and clinical utility were calculated and converted into qualitative grades as suggested previously [36]. Where necessary, the false discovery rate for multiple comparisons was controlled with a two-stage step-up method by Benjamini, Krieger, and Yekutieli [37]. *p* < 0.05 was considered to indicate a statistically significant difference.

## 3. Results

### 3.1. Immune Subtyping of the TCGA Cohort

**Figure 1:**
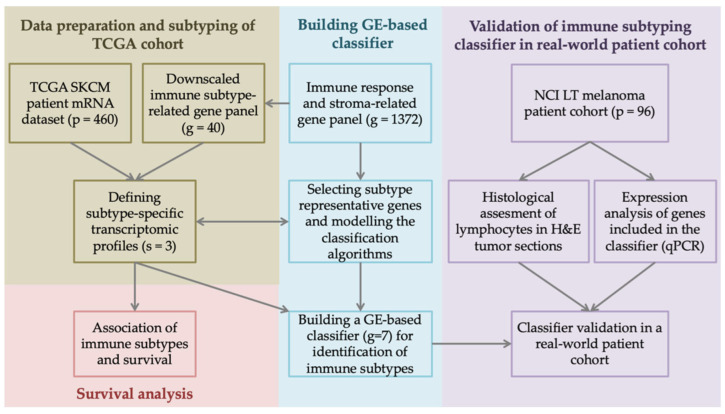
**Study design and analysis workflow.** Clinical and tumor transcriptome data of 480 skin cutaneous melanoma (SKCM) patients were retrieved from The Cancer Genome Atlas (TCGA) database through cBioportal. From an extensive list of 1372 genes coding for immune response and stroma elements, we selected forty genes of interest, which served as a basis for defining subtype-specific transcriptomic profiles and assigning tumors into distinct immune subtypes. Differential gene expression (GE) and survival analysis revealed subtype representative genes, which we further used to build an immune subtyping classifier. The classifier’s performance was validated in a 96 melanoma patient cohort from the National Cancer Institute, Lithuania (NCI LT), and compared with tumor histological and GE profiles. qPCR—quantitative polymerase chain reaction, H&E—hematoxylin–eosin staining.

The workflow of this study is shown in Figure 1. To inspect the immune microenvironment-related transcriptional profiles, we first analyzed the TCGA SKCM dataset, which contains clinical data of patients diagnosed with cutaneous melanoma as well as their bulk tumor RNA-Seq data.

The presence of TILs in tumor parenchyma or stroma can influence the cellular and molecular tissue characteristics and can therefore be reflected in the bulk transcriptomic profile, as demonstrated previously [38,39]. For exploring the patterns in the training TCGA dataset of 460 patients, we assembled a list of 1372 gene entries covering the cancer-immunity cycle and, in particular, focusing on the immune response and stroma elements.

**Figure 2:**
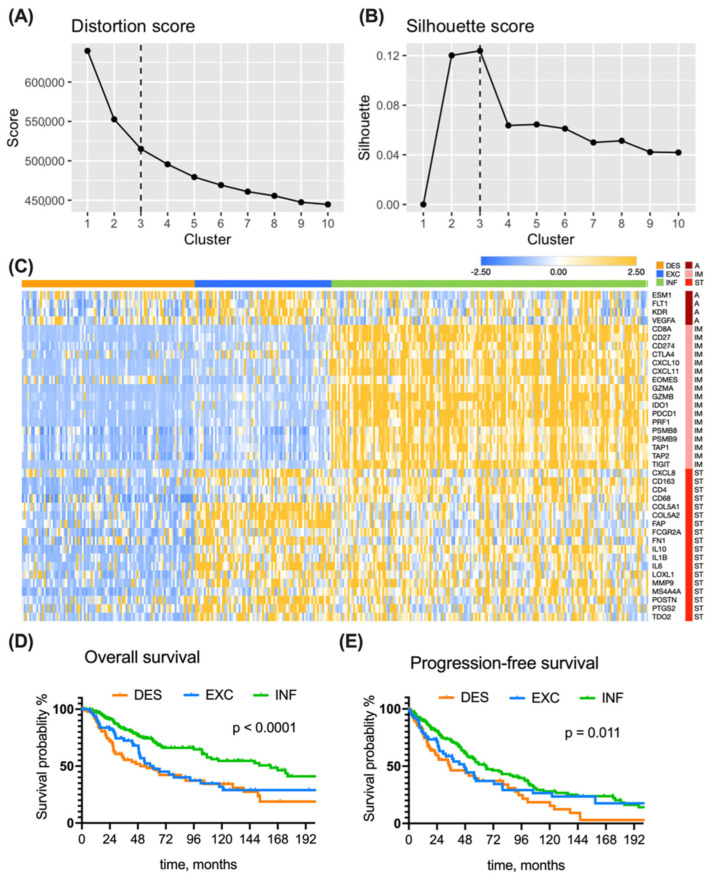
**Subtype discovery in TCGA dataset**. An optimal number of clusters (*k* = 3) was determined from an elbow graph (**A**), where distortion for each *k* was plotted against cluster number, and silhouette plot (**B**), reflecting the quality of fit cohesion and separation. (**C**) A heatmap presenting the expression of genes (rows) in 346 patients (columns). Modified *Z*-score transformed GE levels, depicted as bars of different color intensity, served as a basis for assembling tumors into inflamed (176 patients), immune excluded (75 patients), and immune desert (95 patients) subtypes using the predefined rules and *k*-means clustering. A—angiogenesis, IM—immune response, ST—stroma. (**D**) Kaplan—Meier overall survival curves of TCGA melanoma patients grouped into three immune subtypes. (**E**) Kaplan—Meier progression-free survival curves of TCGA melanoma patients grouped into three immune subtypes. Survival curves were compared with the log-rank test. Group abbreviations: DES—desert, INF—inflamed, EXC—excluded.

To decide on the optimal number of transcriptional clusters, we applied the *k*-means algorithm to the full TCGA dataset with the number of clusters varying from 1 to 10. After plotting the distortion (Figure 2A) and silhouette (Figure 2B), the *k* = 3 cluster number returned the most favorable scores and was therefore selected as an optimal cluster number, also supporting the initial three immune subtype hypothesis.

To highlight the fundamental TME differences among immune subtypes, we exploited a reported signature [14,23] of forty genes codings for relevant elements of angiogenesis, immune response, and reactive stroma. We performed cluster analysis using both *k*-means and previously developed rules-based clustering for immune subtyping [14]. Transcriptional profiles of 346 TCGA-SKCM melanoma samples that were assigned into the same immune subtype by both *k*-means and predefined rule clustering methods demonstrated particular subtype-specific trends, as shown in a heatmap of gene expression (GE) (Figure 2C): desert tumors (51%) bear low GE of both immune- and stroma-related genes; excluded tumors (21%) are characterized by high GE of stroma- and angiogenesis-related genes; inflamed tumors (27%) have distinctly high GE of immune-related genes, while their stroma- and angiogenesis-related GEs are rather heterogeneous.

The above findings suggest that three distinct immune subtypes can be identified within melanoma tumors. The cluster annotation from Figure 2C served for further subtype characterization using survival and differential GE analysis.

### 3.2. Clinical Features of the Immune Subtypes in the TCGA Cohort

Next, we examined the baseline clinical characteristics of TCGA SKCM patients within the assigned immune subtype (Table 1).

The differences in TNM stage and Breslow depth highlighted the statistically significant trends of smaller values in inflamed tumors, which was taken into account during multivariate Cox regression analysis for survival (Table 2).

Interestingly, the distribution of oncogenic mutations in *BRAF*, *NRAS*, and *KIT* (usually mutually exclusive) followed specific patterns. Alterations in BRAF and KIT were most common in excluded tumors. The NRAS mutation was most frequently found in desert tumors. Alterations in KIT were not found in inflamed tumors. Overall, nearly half of the patients in the desert or inflamed groups were wild type for specific mutations, whereas two-thirds of patients with excluded tumors had at least one oncogene mutated.

Although the gender composition among different subtype groups was the same, the other sociodemographic variables (age and BMI) of patients differed.

No significant differences were observed when comparing the Clark level or tumor mutational burden (TMB). Although high TMB in theory could be a prerequisite for TIL attraction and formation of inflamed TME, the two-fold difference in medians of inflamed versus desert tumors does not reach the statistical significance level.

We found a significant difference in overall survival among the patients bearing different tumor subtypes (*p* < 0.0001) (Figure 2D). This was also true at each TNM stage level. Patients from the inflamed group survived longest, with an mOS of 162 months (Table 2), which is nearly threefold more than the mOS of excluded and desert subtypes.

Moreover, the inflamed subtype-bearing melanoma patients were more likely to survive for 3 or 5 years. Among non-inflamed tumors, patients from the excluded group have a better 3-year OS than the desert group; however, at the 5-year cutoff, the proportion of patients at risk is the same (around 50%) in both desert and excluded groups. Cox regression analysis confirmed that patients with non-inflamed tumors have higher risk of death (for desert: unadjusted HR = 2.38, *p* = 0.002, adjusted HR = 1.57 *p* = 0.056; for excluded: unadjusted HR = 1.81, *p* = 0.004, adjusted HR = 1.62, *p* = 0.036) when compared to the inflamed subtype.

Progression-free survival was also different between the three subtypes (Figure 2E). Patients with inflamed tumors had a mPFS of 66 months, which is almost two-fold longer in comparison to patients from the desert group (mPFS = 35 months). Patients with excluded tumors have s significantly higher risk of progression in comparison to inflamed tumors (unadjusted HR = 1.19, *p* = 0.012, adjusted HR = 1.56, *p* = 0.041).

Taken together, the results of the clinical feature and survival analysis highlight the prognostic significance of immune tumor subtyping and its potential clinical utility. These findings encouraged us to develop a practical auxiliary tool for determining the tumor immune subtype.

### 3.3. Building the Immune Subtyping Classifier

**Figure 3:**
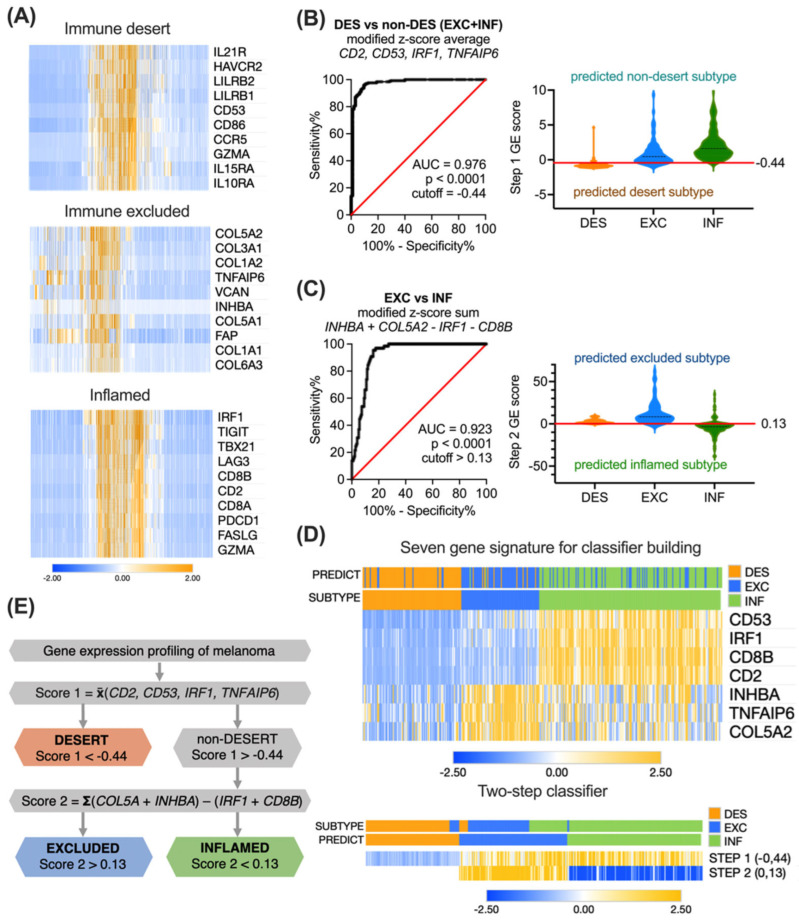
**Development of gene expression score-based algorithm for immune subtyping.** (**A**) Heatmaps showing the modified z-score transformed expression of the top 10 differentially expressed genes in each TCGA subtype cluster. (**B**) Performance of a four-element combination, constructed as a modified z-score average of *CD53*, *TNFAIP6*, *CD2*, and *IRF1* expression, was evaluated by ROC analysis, and a cutoff of −0.44 was selected for distinguishing desert and non-desert subtypes. (**C**) Performance of a four-element combination, constructed as a modified z-score sum *COL5A2* + *INHBA* − *CD8B* − *IRF1*, was evaluated by ROC analysis, and a cutoff of 0.13 was selected for distinguishing excluded and inflamed subtypes. (**D**) Heatmaps presenting the expression of the seven most representative genes alone or in a classifier (rows) in 346 TCGA pretreatment patients (columns) with annotated or predicted clusters. (**E**) A sequence for gene expression score-based immune subtyping of inflamed, excluded, and desert tumors. AUC—area under curve, DES—desert, EXC—excluded, INF—inflamed.

For constructing a feasible classifier that is able to accurately distinguish desert, excluded and inflamed tumors, we decided to narrow down the GE signature by exploring the subtype-specific genes. We applied the differential GE analysis algorithms to the initial 1372 gene list in the TCGA cohort. For each immune subtype, the top 10 genes discriminating the subtype from among the others were picked (Figure 3A).

For excluded and inflamed tumors, these top differentially expressed genes represented the increased GE of stroma- or immune response-related related processes, respectively. In contrast, for desert tumors, the top differentially expressed genes represented the decreased GE of immune response-related pathways and were partially overlapping with inflamed subtype-specific genes.

We next explored the discrimination performance of individual genes and their various combinations using ROC analysis. We aimed at selecting a combination with the least factors and maximal separation efficiency. However, these criteria were not satisfactorily met with a single gene signature, as the investigated immune subtypes are defined not only by the sole quantitative presence of TILs but also by their location in the tumor. Therefore, we hypothesized that a two-step classifier would potentially perform better.

To test this hypothesis, we first decided to determine the presence/absence of TILs to be able to discriminate between the desert and non-desert subtypes. A four-element combination Score 1, constructed as a modified z-score average of desert subtype-specific gene *CD53*, excluded subtype-specific gene *TNFAIP6*, and two inflamed subtype-specific genes *CD2* and *IRF1*:

(1) $$\;\mathrm{Score}\;1=\frac{z\left(CD53\right)+z\left(TNFAIP6\right)+z\left(CD2\right)+z\left(IRF1\right)}{4}$$

yielding the highest attainable AUC = 0.976 (Figure 3B), and with a cutoff of −0.44, demonstrated 96.0% (CI 92.8–97.8%) sensitivity and 90.5% (CI 83.0–94.9%) specificity. The overall accuracy was 94.5% (excellent), the positive clinical utility index was 0.809 (good), and the negative clinical utility index was 0.925 (excellent).

Next, to discriminate between excluded and inflamed tumors in the previously sorted non-desert tumors, we built another four-element combination Score 2 by subtracting the modified z-score sum of two inflamed subtype-specific genes *CD8B* and *IRF1* from the modified z-score sum of two excluded subtype-specific genes *COL5A2* and *INHBA*:

(2) $$\mathrm{Score}\;2\;=z\left(COL5A2\right)+z\left(INHBA\right)-z\left(CD8B\right)-z\left(IRF1\right)$$

This algorithm yielded an AUC = 0.923 (Figure 3C), and with a cutoff of 0.13, it demonstrated 96.9% (CI 89.5–99.5%) sensitivity and 78.4% (CI 71.8–83.4%) specificity. The overall accuracy was 80.4% (good), the positive clinical utility index was 0.550 (fair), and the negative clinical utility index was 0.730 (good).

In total, seven genes involved in two sequential logical steps were combined into a classifier. The clustering of 346 TCGA patients using seven genes, or a two-step classifier resulted in the clearly distinct desert, excluded, and inflamed subtypes (Figure 3D). The overall logic sequence of GE-based immune subtyping of ovarian tumors is summarized in Figure 3E. The accuracy of separation in the TCGA cohort was 83.2% (good) (288 true positives out of 346), with a positive clinical utility index of 0.587 (fair) and a negative clinical utility index of 0.755 (good). The cutoffs are derived from mathematical operations involving z-scores, where a negative value signifies score below the population median, and a positive value indicates score above the median.

**Figure 4:**
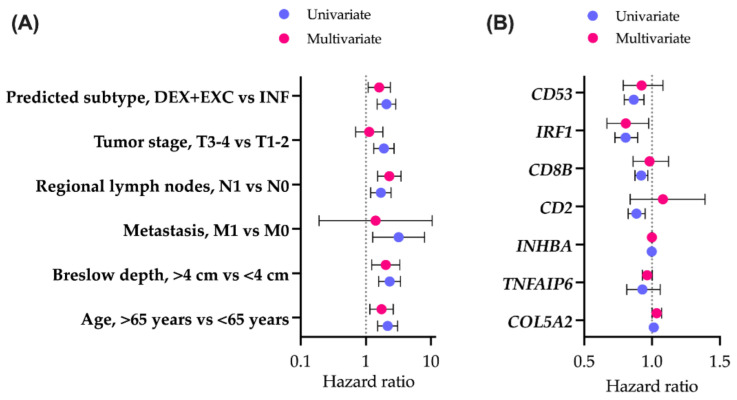
**Overall survival analysis.** Association of immune subtypes and baseline clinical characteristics (**A**) or genes included in classifier construction (**B**) with overall survival. The forest plots indicate the hazard ratios (HRs) and 95% confidence intervals (CIs) for each factor in a univariate and multivariate setting. DES—desert, EXC—excluded, INF—inflamed.

To examine the effect of a created classifier on survival prediction, we performed Cox regression for OS, including other baseline clinical characteristics (Figure 4A).

To compute the exact hazard ratios, excluded and desert groups were pooled into a single group of non-inflamed tumors, as there were no significant differences between their Kaplan–Meier overall survival curves (*p*(adj) = 0.09). Immune subtypes were shown to be associated with survival. Patients bearing non-inflamed tumors had worse prognoses, as indicated by both the univariate and multivariate Cox models (*p* = 0.001 and *p* = 0.018, respectively). As predicted by the GE-based score, patients with inflamed tumors (mOS = 161.7 months, 95% CI 104.9–219.1) survive significantly longer (*p*(log-rank) < 0.001) than patients with non-inflamed tumors (mOS = 58.5 months, 95% CI 43.5–73.5).

Out of seven genes used for subtyping classifier construction, there were three reactive stroma- and four immune response-related elements. All individual immunity-related genes (*CD2*, *CD8B*, *IRF1*, *CD53*) were associated with better prognoses, as reflected in the univariate Cox model (Figure 4B). When combined into a seven-gene classifier, only *IRF1* retained a significant level of association with OS (*p* = 0.027). Individual reactive stroma-related genes (*COL5A2*, *TNFAIP6*, *INHBA*) were not associated with prognosis. In a seven-gene classifier, *COL5A2* was slightly associated with worse survival (*p* = 0.048).

The above results highlight the need for immune subtype-related treatment stratification for melanoma to improve survival, especially for non-inflamed tumors.

### 3.4. Validation of the Immune Subtyping Classifier in a Real-World Patient Cohort

After developing a two-step gene signature-based algorithm for immune subtyping of ovarian tumors in silico, we aimed to analyze its performance in a real-world patient cohort consisting of 96 melanoma patients that underwent primary tumor resection at the National Cancer Institute in Vilnius, Lithuania, in 2018–2020. Patients follow-up continued until October 2023. Due to the heterogeneous nature of our primary patient cohort, consisting of varying disease stages and treatment modalities, statistical control for therapy was not feasible.

**Figure 5:**
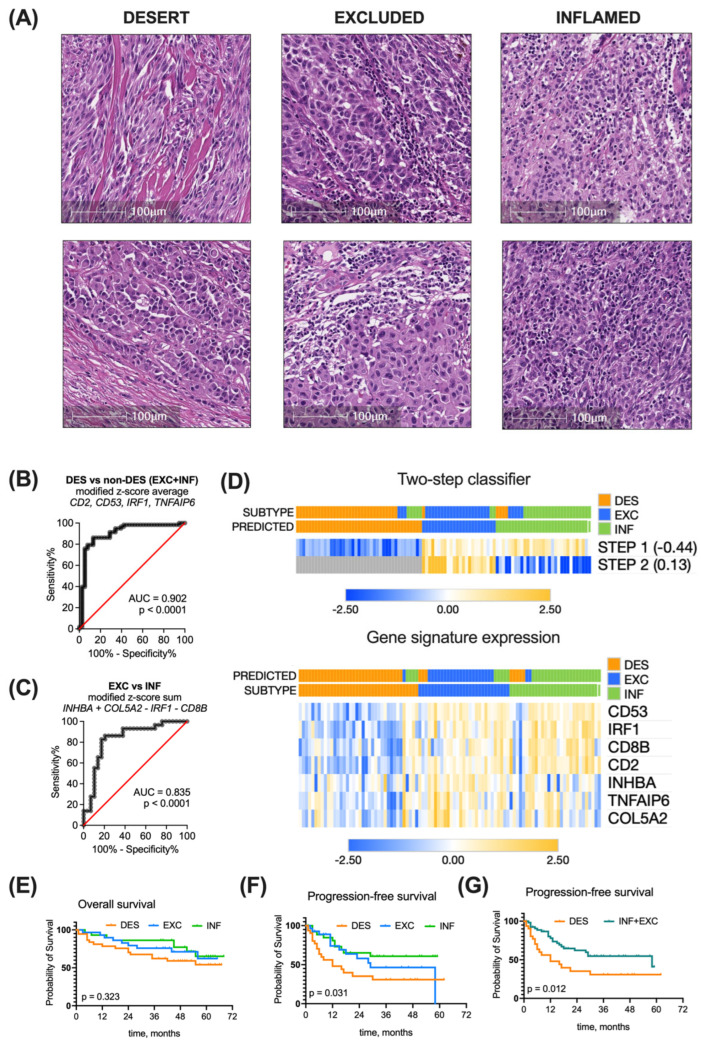
**Immune subtyping of real-world patient cohort.** (**A**). Tumors were classified as inflamed, excluded, and desert by pathological examination of H&E-stained tumor sections. (**B**) Performance of a four-element combination, constructed as a modified z-score average of *CD53*, *TNFAIP6*, *CD2* and *IRF1* expression in discriminating desert and non-desert subtypes, was evaluated by ROC analysis at a cutoff of −0.44. (**C**) Performance of a four-element combination constructed as a modified z-score expression sum *COL5A2* + *INHBA* − *CD8B* − *IRF1* in discriminating excluded and inflamed subtypes was evaluated by ROC analysis at a cutoff of 0.13. (**D**) Heatmaps present the expression of the seven most representative genes alone or in a classifier (rows) in 96 real-world patients (columns) with annotated or predicted clusters. Kaplan–Meier overall survival (**E**), progression-free survival (**F**), and combined progression-free survival (**G**) curves of real-world ovarian cancer patients grouped into different immune subtypes based on the pathological evaluation. Survival curves were compared with log-rank test. AUC—area under curve, INF—inflamed, EXC—excluded, DES—desert.

To determine the immune tumor subtype of the patient cohort, the H&E-stained tumor tissue sections were evaluated by a pathologist for the presence of both intraepithelial and stromal tumor-infiltrating lymphocytes (TILs) (Figure 5A), according to published recommendations of several working groups [29,30]. Based on histological examination of the primary tumor, patients were grouped into the respective immune subtype groups: 40% of patients were attributed to the immune desert group, 30% to the excluded, and 30% to the inflamed group.

In parallel, we measured the relative GE of classifier genes *COL5A2*, *INHBA*, *TNFAIP6*, *IRF1*, *CD53*, *CD8*, and *CD2* in patient tumors using qPCR. We next applied our developed two-step immune subtyping classifier using the modified z-scores of relative GE, which resulted in classifying 43% of patients as the desert subtype, 25% as the excluded subtype, and 32% as the inflamed group. The proportion of this distribution was different than in the initial TCGA cohort (*p* = 0.023).

Classifier-based immune subtyping was compared with the histopathological evaluation. Running step 1 of the classifier (discriminating between desert and non-desert patients) with the determined −0.44 cutoff resulted in AUC = 0.902, 84.5% (CI 73.1–91.6%) sensitivity, and 86.8% (CI 72.7–94.3%) specificity (Figure 5B). The accuracy of this step was 88% (excellent).

Applying step 2 of the classifier (discriminating between excluded and inflamed tumors in the non-desert pool) with the determined 0.13 cutoff resulted in AUC = 0.835, 82.8% (CI 65.5–92.4%) sensitivity, and 82.8% (CI 65.5%#x2013;92.4%) specificity (Figure 5C). The accuracy of this step was 78% (good).

Overall, clustering of the 96 melanoma patient cohort using a developed two-step classifier resulted in 79% accuracy (good)—76 out of 96 tumors were assigned to the same subtype group using both histopathological assessment and the GE-based classifier (Figure 5D).

The survival trends of immune subtype groups in the melanoma patient cohort cannot be directly compared with those of the TCGA cohort due to at least a four-fold longer follow-up time for the latter. For the overall survival of the melanoma patient cohort, the median cannot be determined yet (Figure 5E). As for progression-free survival, the median time to progression in desert subtype-bearing patients was 12 months, in excluded subtype-bearing patients, 29 months, and in the inflamed group, the median was not reached yet. The PFS differs significantly (*p* = 0.0031) (Figure 5F). When we combined the desert and excluded tumors into a single non-inflamed category, a separation of PFS curve months was observed (*p* = 0.012) (Figure 5G), supporting the prognostic significance of immune subtyping.

We also examined the baseline clinical characteristics of the 96 melanoma patient cohort with regard to the assigned immune subtype (Table 3).

We did not observe significant differences when comparing patient age, tumor stage (including all TNM elements), Clark level, and Breslow depth. The *BRAF* alteration pattern was similar to the TCGA cohort—the frequency of *BRAF* mutations in desert group was nearly two-fold lower than in the inflamed and excluded groups, where half or more patients had tumors with altered *BRAF.* However, the differences among real-world melanoma cohort subtypes did not reach the statistical significance level (*p* = 0.067).

To sum up, both histopathological assessment of TILs in H&E-stained tumor sections, as well as a GE-based classifier successfully distinguish desert, excluded, and inflamed subtypes among melanoma samples with 79% overlap. A seven-gene-based classifier as a biomarker could assist the biomedical community by accurately translating qPCR results into clinically relevant immune subtype information.

## 4. Discussion

The recent shift in the cancer-immunity paradigm [2,40] convinced the scientific community that the information stored within the immune TME is remarkably valuable and targetable and promoted an ambitious leap in immunotherapy. However, the heterogeneous treatment outcomes once again reminded us how complex and adaptable the nature of the immune system is. A seminal review by Hegde and Chen, consolidating the major challenges of current cancer immunotherapy, spotlighted the understanding of organ-specific immune contexture and maximizing treatment personalization through composite biomarkers as being among the key critical questions [7]. With our study, we addressed both issues by hypothesizing that desert, excluded, and inflamed immune subtypes can be defined in melanoma tumors and immune subtyping can serve as a source of personalized biomarkers. We believed that our findings could contribute to the arising consensus on the significance of intraepithelial/stromal TILs and their application in clinical practice. We took an approach to first analyze TCGA data and filter out the subtype-specific genes for subsequent validation in the clinical melanoma cohort and matching with the histological assessment.

One of the primary outcomes of our study was the recognition of desert, excluded, and inflamed immune melanoma subtypes both transcriptionally in silico, as well as transcriptionally and histologically ex vivo. Different approaches to melanoma subtyping were already reported [41,42], although they were generally based on genomics or cancer cell histology. Wide exploratory studies addressing the immune aspects of melanoma TME were mostly solely computational [43,44,45], yet often meticulous and conclusive. The conventional and more supervised approaches, like ours, taking advantage of publicly available transcriptomic datasets also existed [46,47]; however, they usually carried different subtype annotations for what we believe could be recognized as immune desert, excluded, and inflamed tumors based on their biological information. Reports adopting the desert, excluded, inflamed nomenclature in melanoma were only histology based [48,49,50]. In this setting, our findings combining transcriptome and histology data, although with their own limitations, carry an element of novelty and contribute to the understanding of the tissue-specific immune contexture in melanoma. Our definition of desert, excluded, and inflamed immune subtypes is aligned with related reports on other cancer types, such as ovarian [14,51,52], lung [53,54,55], head and neck, [52,56,57] breast [55,58], bladder [52,53], gastric [52], colorectal [52], hepatocellular [59], pancreatic [60], and renal cancer [61], and contributes to developing agreement on universal immune contexture elements among different cancer localizations.

It is worth mentioning that nearly half of the studies employing the desert, excluded, inflamed nomenclature (as cited in the previous paragraph) also relate the immune subtypes with response to a checkpoint blockade, emphasizing their emerging significance as predictive biomarkers. In our study, we highlighted the prognostic significance of immune subtyping. The association of inflamed tumors with longer overall survival is widely reported and reviewed [62,63], suggesting inflammation as a very generalized biomarker in cancer. Nevertheless, the frequently used term ‘inflamed’ or ‘hot’ tumor conceals a need for treatment personalization through composite biomarkers [7]. On the other hand, the number of papers reporting survival-associated gene signatures is growing exponentially: the PubMed search for “immune AND gene signature AND prognosis AND cancer” returns more than 6500 entries, out of which 80% were published in the last 3 years. Testing, validation, and reproduction of a large number of potential candidates to translate any biologically meaningful biomarkers into a clinical application remain to be addressed with computational AI algorithms.

Our study is not an exception—we constructed the immune subtyping classifier based on the expression of seven genes. Four of them—*CD2*, *CD8B*, *CD53*, and *IRF1*—are associated with the immune response. CD8 is a marker for cytotoxic T cells that execute direct killing of tumor cells when accurately primed with an antigen and activated [64]. CD2 participates in the formation of the immunological synapse between T cells and antigen-presenting cells [65]. Tetraspanin CD53 on the T cell membrane is important for T cell-mediated immunity and its deficiency results in impaired TCR signaling and proliferation [66]. IRF1 regulates the development of Th1 and Tr1 lymphocytes [67] and programs cDC1 dendritic cells to drive antitumor immunity [68]. CD2, CD8, and CD53 are mentioned together in a leukocyte infiltration score predicting melanoma patient prognosis [69]. SOX10-IRF4-IRF1 axis serves as a potential target to immunologically warm-up melanoma with a “cold” TME [70].

Three other genes—*COL5A2*, *INHBA*, and *TNFAIP6*—represent the reactive stroma compartment in our classifier. None of these genes were previously reported to be associated with melanoma. Inhibin β-a, encoded with *INHBA*, is a member of the TGFβ superfamily and is a marker of reduced survival and poor prognosis in cervical cancer [71]. In our melanoma cohort, we did not observe an association with survival. However, the tumor stroma and extracellular matrix can contribute to retaining otherwise primed and functional T cells and impairing the antitumoral immune response [72]. This phenomenon is not widely reviewed in melanoma, leaving space for further investigation. Interestingly, we have previously reported *CD2* and *COL5A2* in a single gene signature for immune subtyping of desert, excluded, and inflamed ovarian tumors [14], suggesting that underlying biological mechanisms behind immune TME formation can be at least in part shared among tumors in different organs.

We also report the different distributions of major oncogenic melanoma alterations (BRAF, NRAS, KIT) among different immune subtype groups. The *NRAS* mutation was more frequent in the immune desert subtype. The *BRAF* mutation was more characteristic of excluded and inflamed subtypes, as confirmed in both TCGA and patient cohorts. Almost none of the inflamed tumors had alterations in *KIT*. Although the underlying biological meaning and significance of this phenomena are not clear, it might affect combinational treatment (targeted therapy + immunotherapy) decisions [73,74]. For example, in colorectal cancer, the distinct pattern of immune TME in BRAF-mutated tumors suggested the rationale for using checkpoint inhibitors in this subgroup of patients [75].

The paradigm of the desert, immune, and inflamed immune subtypes does not rule out other possible molecular or cellular tumor classification systems, but rather informs about the presence or absence of TILs within different tumor compartments and gives a hint about the character of prevalent immune TME. The supervised discrimination of merely three immune subtypes in our and other studies is to some extent simplified and biased due to its hypothesis-driven nature. Our study design was also inclined to look for distinct transcriptomic profiles, and we discarded a substantial proportion of TCGA entries due to discrepant results using different clustering algorithms. Nevertheless, this approach allowed us to define subtype-specific genes and translate them into a real-world cohort, where we reached 79% overlap between histological assessment and qPCR analysis

While utilizing gene expression values as biomarkers presents some inherent limitations (context dependence, dynamic nature, and complexity of interpretation), normalization strategies help to minimize these challenges. We employed the modified z-score, which is more robust and less affected by a sample size than a standard z-score, yet still can be easily combined to better reflect the respective pathway activity [76]. The combined z-score is also acknowledged as one of several reliable methodologies for scoring individual samples and integrating new patients post classifier development [26]. Looking ahead, the integration of machine learning holds promise in further refining the utilization of gene expression values as reliable biomarkers.

We believe that despite the acknowledged limitations, our findings could advance the research in the immune TME and immunotherapy biomarker field. One of the key strengths of our research lies in its robust methodology and validation in real-world melanoma tumors, which demonstrated a commendable accuracy of 79%. Moreover, our findings have practical implications for personalized medicine approaches in melanoma treatment. By stratifying patients based on immune subtypes, we may be able to tailor therapeutic interventions more effectively. Our results underscore the potential of gene expression-based algorithms as important tools for evaluating the tumor immune microenvironment in clinical practice. Future research could focus on refining our algorithm and validating it on a larger scale to determine its clinical utility and performance in immunotherapy-treated patients.

## 5. Conclusions

Immune tumor subtyping into the desert, excluded, and inflamed groups can provide valuable clues about the prevalent immune TME. We created and validated a simple classifier for reliable immune tumor subtyping with the input of qPCR-derived relative expression levels of seven genes, representing the balance between immune response and reactive stroma in the TME. This tool could serve as an auxiliary means to conveniently obtain clinically relevant information.

## Figures and Tables

**Table 1:** Baseline clinical characteristics of patients in TCGA dataset (N = 346).

Feature	Desert	Excluded	Inflamed	*p*-Value
**N**	95	75	176	
**Age, median (range)**	58 (15–86)	62 (20–90)	56 (18–90)	0.031
**BMI, median (range)**	26.1 (17.8–42.5)	27.3 (17.6–55.5)	28.7 (18.3–49.1)	0.045
**TMB mut/Mb, median (range)**	12.3 (0.6–129.9)	16.4 (0.3–102.2)	25.2 (0.2–1060.3)	0.403
**Stage**				0.003
I	8 (9%)	10 (14%)	40 (25%)	
II	41 (47%)	34 (48%)	46 (29%)	
III	33 (38%)	23 (32%)	68 (42%)	
IV	5 (6%)	4 (6%)	6 (4%)	
28 undocumented				
**Clark level**				0.402
1	2 (3%)	1 (2%)	2 (2%)	
2	2 (3%)	1 (2%)	11 (10%)	
3	9 (13%)	13 (22%)	36 (31%)	
4	44 (62%)	35 (60%)	52 (45%)	
5	14 (20%)	8 (14%)	14 (12%)	
102 undocumented				
**Breslow in mm, median (range)**	5.8 (0.0–75.0)	7.3 (0.5–29.0)	4.2 (0.0–74.0)	0.032
***BRAF* status**				<0.001
wild type	34 (72%)	14 (30%)	56 (47%)	
mutated	13 (28%)	32 (70%)	63 (53%)	
134 undocumented				
***NRAS* status**				0.049
wild type	25 (53%)	33 (72%)	87 (72%)	
mutated	22 (47%)	13 (28%)	22 (28%)	
134 undocumented				
***KIT* status**				0.010
wild type	42 (89%)	39 (85%)	116 (98%)	
mutated	5 (11%)	7 (15%)	3 (2%)	
134 undocumented				

*p*-values were calculated using the χ2 test (categorical variables) and one-way ANOVA or Kruskal–Wallis test (numeric variables). BMI—body mass index, TMB—tumor mutational burden.

**Table 2:** Uni- and multivariate Cox regression analysis of the prognostic relevance of immune subtypes in the TCGA patient cohort.

Group	mOS, Months (95% CI)	Estimated Survival, % (95% CI)		Univariate Analysis		Multivariate Analysis *	
				HR (95% CI)	*p*_adj_	HR (95% CI)	*p*_adj_
**OVERALL SURVIVAL**							
		**3 years**	**5 years**				
INF	162.0 (72.8–133.5)	82.7 (77.4–90.0)	73.4 (66.7–81.9)	1.00 (ref.)		1.00 (ref.)	
EXC	61.2 (37.1–85.4)	72.4 (62.5–85.7)	50.1 (37.2–64.7)	1.81 (1.20–2.72)	0.004	1.62 (1.03–2.57)	0.036
DES	55.6 (23.1–87.9)	57.2 (46.5–69.7)	48.2 (36.7–60.8)	2.38 (1.60–3.41)	0.002	1.57 (0.97–2.55)	0.056
**PROGRESSION-FREE SURVIVAL**							
		**3 years**	**5 years**				
INF	65.9 (47.6–84.3)	72.2 (64.0–78.8)	51.5 (42.2–60.0)	1.00 (ref.)		1.00 (ref.)	
EXC	47.1 (32.8–61.5)	58.8 (44.4–60.6)	37.2 (23.5–50.9)	1.19 (0.81–1.72)	0.012	1.56 (1.02–2.38)	0.041
DES	35.0 (8.8–61.2)	46.4 (32.5–59.2)	37.2 (23.9–50.5)	1.68 (1.18–2.39)	0.377	1.13 (0.72–1.78)	0.586

* Multivariate Cox regression analysis accounting for tumor stage (*p* = 0.621), regional lymph nodes (*p* < 0.001), metastasis (*p* = 0.735), Breslow depth (*p* = 0.006), and age (*p* = 0.009) in the survival context. *p*-values were calculated using a two-sided Wald test and adjusted for multiple comparisons. INF—inflamed, EXC—excluded, DES—desert, mOS—median overall survival, CI—confidence interval, HR—hazard ratio.

**Table 3:** Baseline clinical characteristics of National Cancer Institute melanoma cohort (N = 96).

Feature	Desert	Excluded	Inflamed	*p*-Value
**N**	38	29	29	
**Age, median (range)**	72 (36–88)	65 (32–92)	69 (30–88)	0.456
**Stage**				0.985
I	2 (5%)	1 (3%)	2 (7%)	
II	19 (50%)	17 (59%)	14 (48%)	
III	16 (42%)	10 (35%)	12 (41%)	
IV	1 (3%)	1 (3%)	1 (3%)	
28 undocumented				
**Clark level**				0.378
3	9 (24%)	13 (46%)	11 (41%)	
4	22 (60%)	14 (50%)	14 (52%)	
5	6 (16%)	1 (4%)	2 (7%)	
4 undocumented				
**Breslow depth**				0.300
1–4 mm	15 (41%)	13 (50%)	17 (63%)	
>4 mm	22 (59%)	13 (50%)	10 (37%)	
6 undocumented				
***BRAF* status**				0.067
wild-type	28 (74%)	14 (48%)	15 (52%)	
mutated	10 (26%)	15 (52%)	14 (48%)	

*p*-values were calculated using the χ2 test (categorical variables) and one-way ANOVA (age).

## Data Availability

The data presented in this study are available on request from the corresponding author. The data are not publicly available due to privacy restrictions of healthcare data.
